# Non-culprit ruptured vulnerable plaque healing and stabilization by an aggressive lipid-lowering therapy

**DOI:** 10.1007/s10554-021-02198-z

**Published:** 2021-03-01

**Authors:** Keisuke Shoji, Noriyuki Wakana, Kan Zen, Satoaki Matoba

**Affiliations:** Kyoto Prefectural University of Medicine, Kyoto, Japan

An 80-year-old man with ST-segment elevation myocardial infarction underwent primary percutaneous coronary intervention (PCI) for 99% stenosis of the proximal right coronary artery. He underwent a successful PCI with drug-eluting stent implantation under the guidance of near-infrared spectroscopy intravascular ultrasound (NIRS-IVUS). However, non-culprit ruptured plaques were identified distal to the culprit lesion (Fig. 1A). The maximum 4-mm lipid core burden index (maxLCBI_4 mm_) of the lesion was 743. Moreover, optical coherence tomography (OCT) revealed a disrupted fibrous cap with a residual lipid-rich plaque (LRP). The minimum lumen area (MLA) was 4.4 mm^2^ (Fig. 1A). An aggressive lipid-lowering therapy (10 mg rosuvastatin, 10 mg ezetimibe, and proprotein convertase subtilisin/kexin type 9 (PCSK9) inhibitor) lowered the low-density lipoprotein cholesterol levels from 171 to < 17 mg dL^−1^. One-year follow-up using NIRS-IVUS and OCT revealed a significant maxLCBI_4 mm_ decrease (126), a minimum fibrous cap thickness increase, disrupted fibrous cap disappearance, and ruptured plaque healing with an expanding MLA (10 mm^2^) (Fig. 1B).

**Fig. 1:**
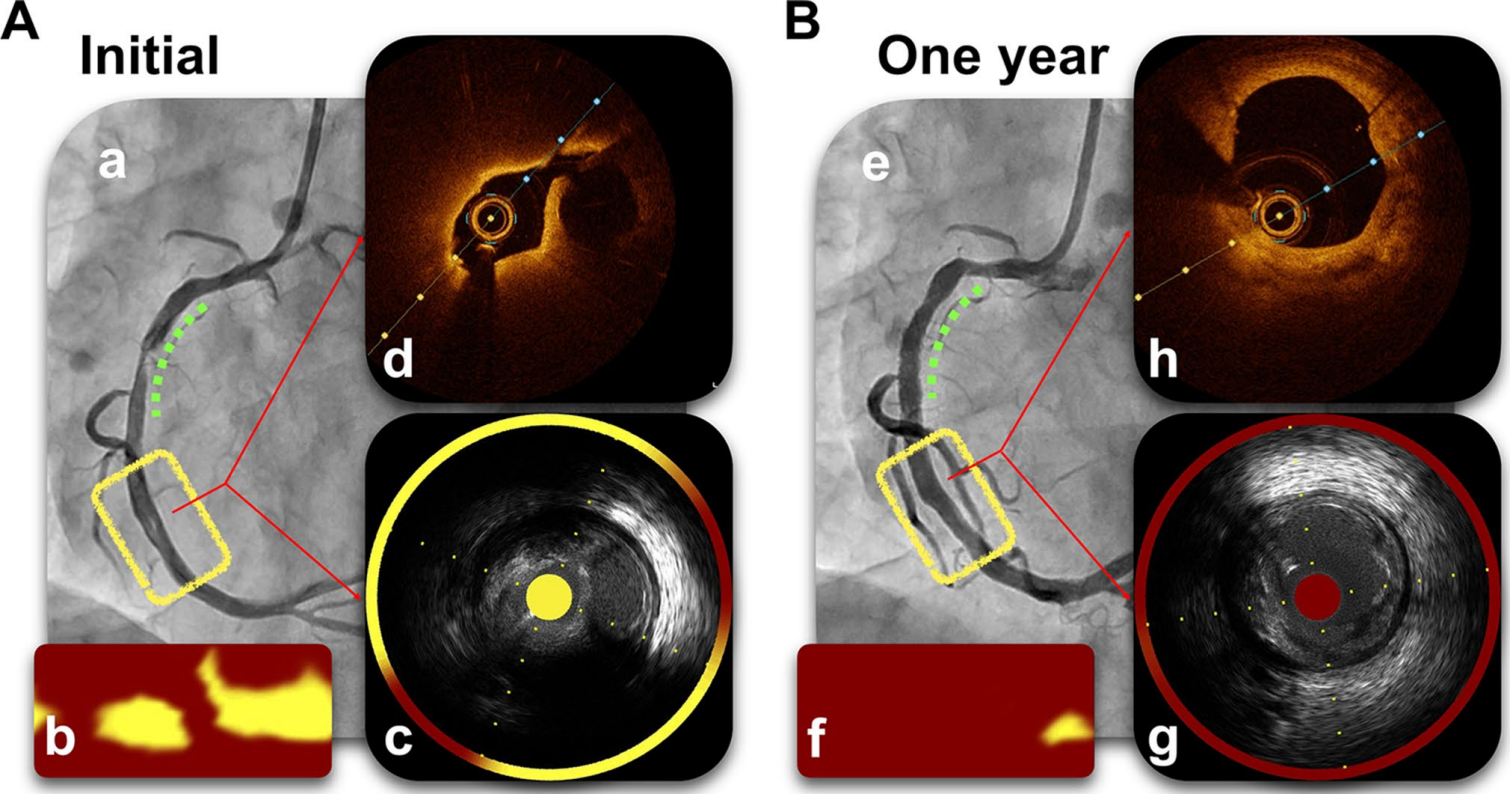
Drastic changes in the coronary images of a ruptured non-culprit lesion. **A** Initial coronary images: **a** coronary angiography, **b** and **c** initial NIRS-IVUS images, and **d** Initial OCT image of the non-culprit lesion. **B** One-year follow-up coronary images: **e** follow-up coronary angiography, **f** and **g** follow-up NIRS-IVUS images, and **h** follow-up OCT image of the non-culprit lesion. Green dotted lines denote the culprit lesions; yellow lines denote the non-culprit lesions. The maxLCBI_4mm_ in the non-culprit lesion has significantly decreased in the follow-up NIRS-IVUS analysis (from 743 to 126) (**b**, **f**). The disrupted fibrous cap in the non-culprit lesion has disappeared in the follow-up OCT analysis (**d**, **h**). *maxLCBI*_*4mm*_ maximum 4-mm lipid core burden index, *NIRS-IVUS* near-infrared spectroscopy intravascular ultrasound, *OCT* optical coherence tomography

Previous intravascular imaging studies reported on the presence of plaque ruptures in both culprit and non-culprit lesions in patients with acute coronary syndrome (ACS). [1, 2] Non-culprit plaque ruptures were associated with a fibroatheroma comprising a residual necrotic core. However, there were no major adverse events in patients treated with medical therapy, including statins [1]. In contrast, subclinical ruptured plaques were associated with a high rate of 1-year revascularization [2].

In our patient, NIRS-IVUS and OCT revealed morphological details and drastic changes of the ruptured non-culprit plaque with a residual LRP. A combination of an aggressive lipid-lowering therapy, consisting of a strong statin and a PCSK9 inhibitor, might have healed and stabilized the non-culprit vulnerable ruptured plaques, without significant stenosis.

These imaging findings support the possibility of administering lipid-lowering therapy for the healing and stabilization of non-culprit ruptured plaques and provide historical evidence for its clinical benefits.

## Data Availability

Not applicable.
